# Molecular Detection of Dengue and Malaria Parasites in Field-Collected Mosquitoes from Meta, Colombia: Implications for Vector-Borne Disease Surveillance

**DOI:** 10.3390/epidemiologia7030076

**Published:** 2026-06-01

**Authors:** Carolina Hernández, David Martinez, Marcela Montilla, Marina González-Robayo, Norma Pavas-Escobar, Plutarco Urbano, Omar Cantillo-Barraza, Davinzon Martínez, Catalina Ariza, Luz Helena Patiño, Juan David Ramírez, Liliana Sánchez-Lerma

**Affiliations:** 1 Centro de Investigaciones en Microbiología y Biotecnología-UR (CIMBIUR), School of Sciences and Engineering, Universidad del Rosario, Bogotá 111221, Colombia; dcahernandezc@gmail.com (C.H.); davidf.martinez@urosario.edu.co (D.M.); luzh.patino@urosario.edu.co (L.H.P.);; 2 Laboratorio de Salud Pública, Secretaría de Salud Departamental Meta, Villavicencio 500001, Colombianorma.pavas@gmail.com (N.P.-E.); 3 Facultad de Medicina, Universidad Cooperativa de Colombia, GRIVI, Villavicencio 500001, Colombia; 4 Grupo de Investigaciones Biológicas de la Orinoquia, Universidad Internacional del Trópico Americano (Unitrópico), Yopal 850001, Colombia; plurbanus@gmail.com (P.U.); davinsonmartinez9@gmail.com (D.M.);

**Keywords:** arbovirus, dengue virus, *Culex*, *Aedes*, entomo-virological surveillance, entomological surveillance, rural area

## Abstract

Background/Objectives: Vector-borne diseases (VBDs) remain a major global public health challenge, particularly in tropical and subtropical regions. In eastern Colombia, the department of Meta reports a high incidence of arboviral infections such as dengue, as well as parasitic diseases including malaria and leishmaniasis. This study aimed to conduct baseline entomological surveillance and molecular screening of Diptera vectors to detect the circulation of arboviruses and parasitic pathogens in two municipalities of Meta, Fuente de Oro and Vista Hermosa. Methods: Adult mosquitoes and sand flies were collected in both municipalities and identified primarily at the genus level, with *Anopheles* specimens identified to species level. A total of 790 insects were collected, of which 780 were processed in 148 pools and 10 were analyzed individually. Molecular detection of pathogens was performed using PCR and RT-PCR to screen for dengue virus (DENV) serotypes, Zika virus (ZIKV), Chikungunya virus (CHIKV), Oropouche virus (OROV), *Plasmodium* spp., and *Leishmania* spp. Results: DENV was detected in 34.8% (55/158) of the processed pools, with DENV-1 identified as the most prevalent serotype. *Culex* was the most abundant genus overall, particularly in Fuente de Oro, while *Aedes* predominated in Vista Hermosa. MIR estimates indicated higher molecular detection likelihood in *Aedes* compared with *Culex*. *Plasmodium vivax* and *P. falciparum* were detected in pools of *Anopheles darlingi* and *Anopheles rangeli*, respectively. No molecular evidence of *Leishmania* DNA was detected in *Lutzomyia* specimens, and no positive detections were observed for ZIKV, CHIKV, or OROV. Conclusions: The molecular detection of DENV and *Plasmodium* spp. in field-collected vectors provides valuable baseline evidence of pathogen circulation in Meta, Colombia. While the findings do not imply vector competence, they highlight the importance of sustained entomological surveillance to inform integrated vector control strategies and guide future studies incorporating species-level identification and longitudinal sampling in endemic regions.

## 1. Introduction

Vector-borne diseases (VBDs), caused by bacteria, parasites, and viruses, represent a major global public health challenge, accounting for approximately 17% of all infectious diseases and an estimated 700,000 deaths annually. The burden of VBDs is highest in tropical and subtropical regions, particularly in sub-Saharan Africa and Southeast Asia, although the Americas have experienced a substantial increase in arboviral outbreaks in recent decades, including dengue, chikungunya, and Zika since 2014 [1]. These arboviral diseases are primarily transmitted by mosquitoes of the family Culicidae, notably *Aedes aegypti* and *Aedes albopictus* [2,3,4]. Beyond arboviruses, parasitic VBDs also contribute significantly to global morbidity and mortality. Malaria, transmitted by *Anopheles* mosquitoes, was responsible for approximately 597,000 deaths in 2023 according to the World Malaria Report 2024 [5], while leishmaniasis, transmitted by phlebotomine sand flies, remains a major public health concern in many endemic regions [6]. The broad geographic distribution and hematophagous behavior of these vectors facilitate pathogen transmission across both rural and urban environments [7].

Molecular biology techniques, including PCR and RT-PCR, have long been integral to entomological surveillance in endemic settings, enabling sensitive and specific detection of pathogens circulating in vector populations [8]. These approaches have identified arboviruses in mosquito species that are not considered primary vectors, underscoring the complexity of arbovirus ecology and the potential involvement of multiple vector species in maintaining transmission cycles [9]. While surveillance efforts have historically focused on *Aedes aegypti* and *Aedes albopictus* as the principal vectors of dengue virus (DENV), other mosquito genera—such as *Culex* (subgenera *Culex* and *Melanoconion*), *Haemagogus*, *Sabethes*, *Coquillettidia*, *Mansonia*, and *Psorophora*—have been implicated as potential vectors, although their vectorial capacity remains incompletely understood [10,11,12,13]. In parallel, sustained entomological surveillance is also critical for parasitic VBDs, particularly malaria, where elimination and control programs have been implemented in several countries. In this context, entomological monitoring plays a fundamental role in identifying vector species and the *Plasmodium* parasites they harbor [14].

In Colombia, entomological studies conducted in coastal ecosystems of the Caribbean region have detected several arboviruses in *Culex* mosquito pools, including St. Louis encephalitis virus (SLEV), West Nile virus (WNV), Venezuelan equine encephalitis virus (VEEV), and *Culex* flavivirus (CxFV), as well as VEEV in pools of *Psorophora* mosquitoes [2]. Additionally, multiple studies have documented the molecular detection of *Plasmodium* spp. and *Leishmania* spp. in insect vectors collected in endemic regions of the Atlantic, Pacific, and Amazon areas of Colombia [15,16,17,18,19,20]. These investigations have largely relied on molecular methods, which offer improved sensitivity and specificity over traditional techniques and enable the molecular detection in *Anopheles* mosquitoes and phlebotomine sand flies [15,16,17,18,19,20].

Despite this progress, other endemic regions of Colombia, such as the Orinoquia, remain understudied with respect to entomological surveillance [21,22]. This region reports a high incidence of medically important arboviruses, including DENV, as well as parasitic infections such as malaria and leishmaniasis [21,22]. However, studies aimed at detecting the pathogens responsible for these diseases in insect vectors from the Orinoquia are scarce.

Given the field-based nature of this study, mosquitoes were identified primarily at the genus level and analyzed in whole-body pools. Accordingly, the objective of this work was to provide baseline molecular evidence of pathogen circulation rather than species-specific assessments of vector competence or transmission risk. Specifically, we screened insect vectors from the department of Meta, one of Colombia’s main endemic foci, to detect arboviruses and the pathogens that cause malaria (*Plasmodium* spp.) and leishmaniasis (*Leishmania* spp.). Within Meta, the municipalities of Fuente de Oro and Vista Hermosa were selected due to their high reported incidence of dengue and malaria cases [23].

## 2. Materials and Methods

### 2.1. Dipteran Sampling and Collection Area

**Figure 1:**
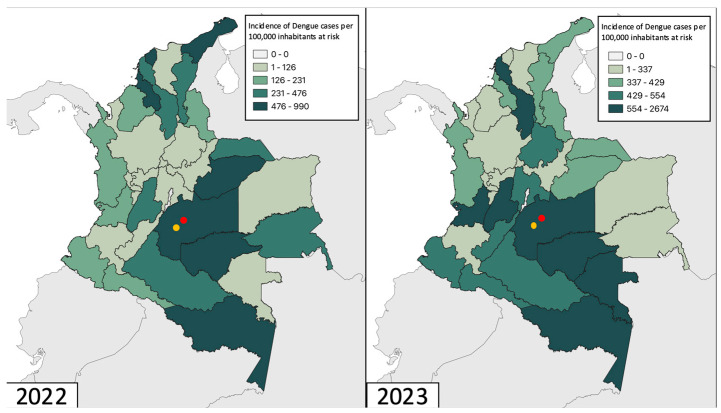
Dengue incidence in Colombia during 2022–2023 and study sites. Map of Colombia, showing Dengue incidence data (reported cases per 100,000 population at risk). The sampled municipalities are highlighted with a red dot Fuente de Oro and the yellow dot indicates Vista Hermosa. The data used for these maps were obtained from Colombia’s Public Health Surveillance System (Sivigila) (Instituto Nacional de Salud, 2022–2023).

Diptera collection was carried out in two different municipalities (Fuente de Oro and Vista Hermosa) in the department of Meta, Colombia, one of the departments most affected by dengue during the last epidemic cycle in 2023, with an incidence of 1560 cases per 100,000 population at risk for Dengue (Figure 1). Sampling was conducted at selected sites and time points, providing a snapshot of pathogen circulation rather than continuous or longitudinal coverage. The municipality of Fuente de Oro has a total area of 576 km^2^, with 44.4% of the population living in rural areas and an incidence of 5152 [23]. On the other hand, the municipality of Vista Hermosa has a total area of 4749 km^2^, with 56.1% of the population living in rural areas and an incidence of dengue fever of 6207 [23]. Sampling sites were selected based on incidence values and strategic points of mosquito breeding. Non-probabilistic convenience sampling was conducted with the help of the Departmental Secretary of Meta on 31 October, 1 November 2022, and 17 and 18 May 2023 (rainy season). Black light traps were set for 24 h in the selected areas, and/or insects were caught manually during the day with mechanical vacuum cleaners. The most entomologically important insects from the family Culicidae and Psychodidae were selected at each sampling point and grouped into pools of 1 to 10 individuals, based on genus identification through morphological evaluation (Table S1). Insects were identified to the genus level due to logistical constraints associated with field collection, specimen preservation, and processing throughput. While species-level identification provides higher ecological resolution, genus-level identification is commonly used in surveillance studies to detect circulating pathogens across vector groups. Whole-body pools were analyzed to maximize detection sensitivity under field surveillance conditions. As a result, positive molecular detections may reflect the presence of viral RNA from recent blood meals rather than active infection or transmission competence. The Technical Research Committee at the University of Rosario in Bogotá, Colombia, with approval code DVO005 1585-CV1427 on 8 June 2021: “Molecular Surveillance of Vector Borne Diseases (VBDs) and Emerging Diseases in the Orinoco region”.

The samples were preserved in DNA/RNA shield (Zymo. R1100-50) at −4 °C in vials labelled with the coordinates and sampling site. Finally, the samples were transported to the Microbiology Laboratory of the Universidad del Rosario in Bogotá, Colombia, for processing and molecular analysis.

### 2.2. Nucleic Acids Extraction and cDNA Synthesis

Pooled and individual entomological material (Table S1) was homogenized using ZR BashingBead^TM^ lysis tubes (Lysis Tubes-ref. S6003-50) (Zymo Research, Irvine, CA, USA) with 200 μL DNA/RNA shield buffer at 30 rpm for 20 min in the TissueLyser II^®^ tissue homogeniser (Qiagen, Hilden, Germany), followed by centrifugation at 10,000 rpm for 2 min. Nucleic acid extraction was performed from the supernatant obtained in the previous step using the Hamilton Microlab Star automated system and the MagBead Quick-DNA/Viral RNA Kit (Zymo Research, Irvine, CA, USA) according to the manufacturer’s recommended instructions. Once the DNA and RNA was obtained, its concentration and quality were quantified using the Nanodrop-2000 spectrophotometer (Thermo Fisher Scientific, Waltham, MA, USA) and stored at −80 °C. RT-PCR was then performed on the RNA to generate cDNA (complementary DNA) using the LunaScript RT SuperMix Reverse Transcriptase Kit enzyme (NEB #E3010) (New England Biolabs, Ipswich, MA, USA). The cDNA was stored at −30 °C.

### 2.3. Conventional Polymerase Chain Reaction for the Identification of Dengue Virus Serotypes and Plasmodium Species

Detection of DENV serotypes (DENV 1-4) was performed only in the mosquitoes of the genera belonging to the subfamily *Culicinae* by multiplex PCR using previously reported primers for the region of the C-prM gene (Table 1). PCR conditions were performed according to the previously described protocol [24].

For *Plasmodium*, amplification of the 18S ribosomal RNA (18S rRNA) gene was carried out. PCR only identified in mosquitoes of the genus *Anopheles*, reactions were performed in a final volume of 12.5 μL containing 2× GoTaq^®^ Colorless Master Mix (Promega, Madison, WI, USA), 10 μM of each primer (rPLU5 and rPLU6; Table 1) and 0.8 μL of cDNA. The thermal profile consisted of an initial denaturation cycle of 95 °C for 5 min, 25 cycles of 58 °C for 2 min, 72 °C for 2 min, 94 °C for 1 min, and finally a final extension cycle of 72 °C for 5 min. *Plasmodium* positive pools were processed to identify the species, *Plasmodium falciparum* and *Plasmodium vivax*. PCR reactions were performed in a final volume of 12.5 μL containing 2× GoTaq^®^ Colorless Master Mix (Promega, # M7133), 10 μM of each primer (rFAL1/rVIV1 and rFAL2/rVIV2; Table 1) and 0.8 μL of cDNA. The thermal profile consisted of an initial denaturation cycle of 94 °C for 4 min, 35 cycles of 94 °C for 30 seg, 58 °C for 1 min, 72 °C for 1 min, and finally a final extension cycle of 72 °C for 4 min. Visualization of the gene fragments was carried out using 2% agarose gel electrophoresis in 1X TBE buffer and 1 μL of SYBR^®^ Safe (Invitrogen^®^, Carlsbad, CA, USA) as an intercalating agent, then the gel was exposed to UV light to observe the amplification band of each, a band of 208 base pairs (bp) was observed for DENV-1, a band of 119 bp for DENV-2, a band of 288 bp for DENV-3, a band of 260 bp for DENV-4, a band of 1200 pb for *Plasmodium,* a band of 205 pb for *Plasmodium falciparum* and a band of 117 pb for *Plasmodium vivax*.

### 2.4. Real-Time Polymerase Chain Reaction for the Detection of Chikungunya, Zika, Oropouche and Leishmania

Identification of CHIKV, ZIKV, OROV and *Leishmania* was performed by real-time reverse transcription–polymerase chain reaction (qRT-PCR) using the appropriate primers and Taqman probes proposed previously (Table 1). The qRT-PCR enzyme used was PerfeCTa^®^ qPCR ToughMix^TM^, Low ROX^TM^ (Quantabio, Carlsbad, CA, USA). CHIKV, ZIKV y OROV was performed only in the mosquitoes of the genera belonging to the subfamily *Culicinae*. For CHIKV, 10 μM of each primer, 5 μM of probe and 1.2 μL of cDNA were used. The thermal profile consisted of an initial denaturation at 95 °C for 2 min, 40 cycles of 95 °C for 15 s, 55 °C for 45 s. For ZIKV, 10 μM of each primer, 25 μM of the probe and 1.2 μL of cDNA were used. Amplification conditions were: one cycle at 95 °C for 1 min, 40 cycles of 95 °C for 15 s and 56 °C for 1 min. For OROV, 20 μM of each primer, 20 μM of probe and 1.2 μL of cDNA were used. The amplification conditions were: one cycle of 95 °C for 1 min, 40 cycles of 95 °C for 15 s and 59 °C for 1 min. Detection of *Leishmania* was performed only in the insects of the genus *Lutzomyia*, 10 μM of each primer, 5 μM of probe and 1.2 μL of cDNA were used. The amplification conditions were: one cycle of 95 °C for 5 min, 40 cycles of 94 °C for 15 s and 60 °C for 1 min. To perform the real-time PCR, the cut-off Ct value was ≤39 based on several criteria, including the Centers for Disease Control and Prevention (CDC) interpretation criteria, where a sample was considered positive if primer sets showed amplification with cycle threshold (Ct) values ≤ 38.5. Similarly, positive tests were confirmed in duplicate. We used as positive controls Synthethic DNA provided by TWIST and as negative controls, samples previously tested as negative for these microorganisms.

### 2.5. Statistical Analysis

First, the results of DENV molecular detection were analyzed in terms of absolute and relative frequencies for the variables considered in the study (municipalities and vector genus). The frequency of molecular detection was calculated as the minimum infection rate (MIR), the ratio of the number of positive pools to the total number of individuals. The MIR was calculated for each mosquito genus for each of the DENV serotypes. Plots were constructed using the ggplot2 package included in the R software (RStudio Team, Boston, MA, USA, v.5.0), and maps were created using the Orange V3.37 software.

## 3. Results

### 3.1. Insect Diversity and Geographic Distribution

**Figure 2:**
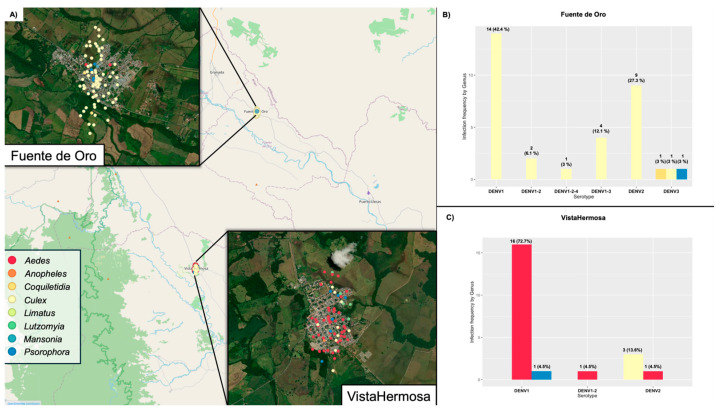
Spatial distribution of insects and the frequency of molecular detection by DENV serotypes. (**A**) Map of the two sampling municipalities, Fuente de Oro and Vista Hermosa, showing the distribution of the captured insects and the frequency of molecular detection by DENV serotypes in (**B**) Fuente de Oro and (**C**) Vista Hermosa.

A total of 790 insects were captured at the two municipalities, of which 780 were processed in pools (*n* = 148) and 10 individually (Table S1). The pools were constructed by genera to assess the circulation of arboviruses and parasites at a general level in the study municipalities, rather than to characterize insect diversity. Eight mosquito genera were identified, *Culex* was the genus found in the highest abundance (51.1% *n* = 404; Table 2) followed by *Aedes* (50 pools, 31.6%), *Psorophora* (12 pools, 7.6%), *Lutzomyia* (7 pools, 4.4%), *Anopheles* (3 pools, 1.9%), *Coquilletidia* (2 pools, 1.3%), *Mansonia* (1 pool, 0.6%), and *Limatus* (1 pool, 0.6%) (Figure 2A and Table S1). By municipality, *Aedes* is the genus found in greatest abundance in Vista Hermosa (96% *n* = 48; Table 2), while in Fuente de Oro, *Culex* is the genus found in greatest abundance (87.8% *n* = 72; Table 2). Vista Hermosa has the highest diversity of mosquito genera captured, with six genera identified and the genera *Anopheles, Limatus* and *Lutzomyia* were found only at this municipality (Table 2).

### 3.2. Arbovirus Detection and MIR of Dengue Viruses

**Figure 3:**
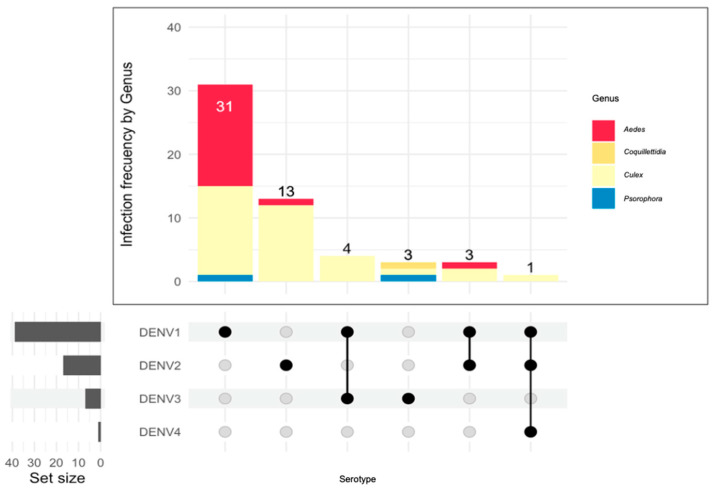
Frequency of molecular detection and co-occurrence by Dengue serotypes in the mosquito genera collected.

Of the 158 pools processed, the overall DENV molecular detection rate was 34.8% (55/158), while no infected pools were found for the other arboviruses (CHIKV, ZIKV, OROV). A higher rate of DENV molecular detection was observed in the genus *Culex*, most of which were found in Fuente de Oro. The most abundant DENV serotype was DENV1, present in 39 pools (24.68%;39/158) (Figure 3), mainly in pools of the genus *Culex* (53.8%;21/39), followed by the genus *Aedes* (43.6%;17/39) and one pool of the genus *Psorophora* (Figure 3). In the *Culex* genus pools all serotypes DENV1 to 4 were identified, whereas in the *Aedes* genus pools only DENV1 and 2 were identified. In the *Psoropohora* genus pools only DENV1 and 3 were identified and in the *Coquilletidia* genus only one pool infected with DENV3 was found (Figure 3). In the two municipalities of Fuente de Oro and Vista Hermosa, the serotype with the highest molecular detection rate was DENV1, but in Fuente de Oro it was found only in individuals of the genus *Culex* (21/33), whereas in Vista Hermosa it was found in the genus *Aedes* (17/22) and in the genus *Psorophora* (1/22) (Figure 2). The four serotypes were identified in Fuente de Oro and almost all co-infected pools with more than one serotype: DENV1-2 (1/2), DENV1-3 (4/4) and DENV1-2-4 (1). In Vista Hermosa, on the other hand, only serotypes DENV1 and 2 were identified, and only one pool of the *Aedes* genus was found to be co-infected with these two serotypes (Figure 2). In terms of MIR values, DENV1 has the highest values in the *Culex* and *Aedes* genera, the latter being the highest with a value of 62.96. DENV2 values were higher in the *Culex* genus (37.13) than in the *Aedes* genus. Finally, DENV4 was only identified in the genus *Culex* with a value of 2.48 (Table 2).

### 3.3. Plasmodium and Leishmania Detection

A total of 43 individuals from the genus *Lutzomyia* were captured and processed in seven pools; however, none tested positive for *Leishmania*. On the other hand, 17 individuals of the genus *Anopheles* were captured and processed in 3 pools, two of which were positive for *Plasmodium*. The insects from these two pools were captured in the village of Campo Alegría, in the municipality of Vista Hermosa. One of the pools corresponds to *Anopheles darlingi* with 10 individuals positive for *Plasmodium falciparum* and the other to *Anopheles rangeli* with 4 individuals positive for *Plasmodium vivax*.

## 4. Discussion

The design of effective arbovirus prevention and vector control strategies relies on knowledge of the presence and baseline circulation of pathogens in their vectors and/or potential vectors. Entomovirological surveillance plays a central role in this process by providing essential information on the local transmission of arboviruses. In this study, we performed molecular detection of relevant arboviruses, *Plasmodium*, and *Leishmania* in insects collected from two municipalities, Fuente de Oro and Vista Hermosa, in the department of Meta, Colombia.

In this study, given the field-based nature of this study, we chose to identify insects at the genus level rather than species level due to morphological variability, field-based logistical constraints, and the high ecological redundancy observed among species within the same genus in tropical regions. This approach enables robust characterization of vector communities, particularly when the primary goal is to assess patterns of abundance, distribution, and relative dominance. The *Culex* genus, for instance, comprises numerous species that share key ecological traits such as generalist feeding behavior, tolerance to diverse habitats, and vector competence for pathogens like West Nile virus and Venezuelan equine encephalitis virus [31]. Moreover, previous studies have shown that genus-level trends often mirror epidemiological risk patterns in endemic regions and are commonly used in baseline or exploratory entomological surveillance [32]. Considering these factors—and given that in areas like Fuente de Oro, 44.4% of the population resides in rural settings with limited access to molecular identification tools or trained taxonomists—genus-level identification represents a practical and scientifically valid strategy for initial ecological assessments.

We first identified the genera of captured mosquitoes and found that *Culex* was the most abundant (51.1%; N = 82) (Table 2), representing more than half of all mosquitoes collected. This dominance was especially evident in Fuente de Oro, where 87.8% of mosquitoes belonged to the *Culex* genus. The high prevalence of *Culex* has also been documented in other studies, where species such as *Cx. pipiens* accounted for 75% of all mosquitoes captured [33]. Moreover, that study reported the presence of this species across three habitat types—urban, peri-urban, and rural—supporting the idea that most *Culex* species exhibit generalist behavior and a broad distribution range. This is consistent with our findings, as 44.4% of the population in Fuente de Oro resides in rural areas [34], which comprise diverse habitats (urban, peri-urban, and rural).

The same study also evaluated gene flow in *Cx. pipiens* across different habitats using ten microsatellite markers, revealing extensive gene flow both within and between ecological zones. Notably, urban environments neither promoted nor restricted gene flow among populations [33], suggesting continuous movement of *Culex* mosquitoes across habitats. This observation is relevant because certain *Culex* species have been implicated as vectors of arboviruses such as West Nile virus [3]. Given their high abundance and documented involvement in the transmission of some arboviruses, *Culex* mosquitoes may contribute to the maintenance of arbovirus circulation. However, this potential role remains speculative and requires confirmation through targeted entomovirological studies assessing vector competence and epidemiological relevance.

In our study, molecular detection revealed active circulation of DENV within the sampled communities, with an overall molecular detection rate of 34.8%. DENV1 was the most frequently detected serotype and was predominantly identified in *Culex* pools (53.8%). However, MIR estimates revealed a contrasting pattern: *Aedes* mosquitoes exhibited higher MIR values (MIR-DENV1: 62.96; Table 2) than *Culex* (MIR-DENV1: 51.98; Table 2). This suggests that *Aedes* individuals were more likely to be infected with DENV1 (6.3%), consistent with previous reports from Mexico where MIR-DENV values of up to 125 have been described for *Aedes* spp. [35]. These findings are in line with the well-established role of *Aedes* mosquitoes as the primary vectors of DENV, supported by their strong anthropophilic feeding behavior, in contrast to *Culex* species, which preferentially feed on non-human hosts such as domestic animals [36].

Interestingly, the MIR values observed for *Culex* in this study were higher than those reported in some previous surveillance efforts. For example, MIR values of 1.6 have been documented for *Cx. quinquefasciatus* in Mexico [19], and positive pools of *Cx.* (*Melanoconion*) *vaxus* (DENV2) and *Cx. quinquefasciatus* (DENV4) have been reported in Brazil [21,22]. These differences may partly reflect the inherent limitations of MIR calculations, which are influenced by pool size and composition and are not standardized across studies. As such, MIR values are more appropriately interpreted for within-study comparisons rather than direct cross-study evaluations, although they remain useful indicators of local arbovirus circulation [10,37]. Additional factors, such as differences in viral load or vector–virus interactions, may also influence detection. For instance, studies on Mayaro virus in *Ae. aegypti* have shown that low viral loads can limit molecular detection [38], underscoring the importance of pool composition and sample size.

Despite the lack of conclusive evidence supporting *Culex* mosquitoes as competent biological vectors of DENV, we observed relatively high molecular detection rates and MIR values in *Culex* pools (Table 2). Entomovirological surveillance has historically focused on *Ae. aegypti* and *Ae. albopictus*, while *Culex* species have received comparatively less attention. Experimental studies assessing the vector competence of *Cx. quinquefasciatus* for DENV have reported very low levels of viral replication, suggesting limited capacity for transmission [39]. Nevertheless, emerging evidence indicates that insect-specific viruses may modulate arbovirus molecular detection dynamics [40], and notable differences in microbiota—particularly the virome—have been observed between laboratory-reared and wild mosquito populations [41]. Moreover, field studies from northeastern Brazil have reported natural DENV2 and DENV3 molecular detections in *Cx. quinquefasciatus* pools during outbreaks [42]. While *Culex* species are not considered primary DENV vectors, these observations suggest they may act as incidental hosts or epidemiological indicators of viral circulation in settings where they coexist with established vectors. Collectively, our findings highlight the need for further integrated studies combining species-level identification, tissue-specific analyses, and experimental validation to clarify the role of *Culex* mosquitoes in arbovirus ecology.

With regard to *Leishmania*, no *Lutzomyia* specimens were found to be infected, which contrasts with the reported cutaneous leishmaniasis incidence in Meta (115.88 cases per 100,000 inhabitants at risk) [43]. However, disaggregated data show that the highest burden occurs in southern municipalities bordering Guaviare, the department with the highest incidence in Colombia in 2022 (961.02) [43]. By contrast, our collections were conducted in central Meta, where leishmaniasis prevalence is lower. Additionally, *Lu. gomezi* is the only *Lutzomyia* species reported in Meta, suggesting that either geographical or ecological barriers may limit its distribution into northern municipalities, or that no active transmission cycles were ongoing at the time of collection [43]. Broader and more systematic sampling will be necessary to test these hypotheses.

For malaria, the main Colombian vectors are *An. darlingi*, *An. nuneztovari*, and *An. albimanus*, in which natural detections with *Plasmodium vivax* and *P. falciparum* have been identified in departments such as Chocó, Valle, and Córdoba [44]. Our findings expand this knowledge by documenting 13 individuals (two pools) of *An. darlingi* circulating in Vista Hermosa, with one pool testing positive for *P. falciparum*. Although Meta reports a relatively low malaria incidence (2.7), the detection of this parasite in a primary vector poses a significant risk for local transmission. We also detected *P. vivax* in one pool of *An. rangeli* (four individuals). While considered a secondary vector in Colombia, *An. rangeli* has been reported as naturally infected with *P. vivax* in southern regions [45]. Together with our data, this suggests that non-primary *Anopheles* species should be further investigated to better understand their role in malaria circulation and transmission dynamics.

Our study has several limitations inherent to field-based entomological surveillance that should be considered when interpreting the results. First, the restricted spatial and temporal sampling effort, while valuable for baseline assessment, limits the generalizability of our findings beyond the specific study sites. Second, mosquitoes were identified at the genus level, which reduces ecological resolution and precludes species-specific inferences regarding the role of individual taxa in DENV transmission, particularly within the *Culex* complex.

In addition, molecular analyses were performed on whole-body mosquito pools rather than dissected tissues. Consequently, the detection of viral RNA may reflect recent blood meal ingestion rather than active infection, viral dissemination to salivary glands, or transmission competence. This limitation is further compounded by the absence of pre-processing classification by sex or gonotrophic status (e.g., blood-fed, gravid, unfed), which restricts our ability to distinguish between passive detection of pathogen nucleic acids and biologically meaningful infection. This consideration is especially relevant for *Culex* mosquitoes, which were highly abundant in the study area; therefore, positive detections within this genus should be interpreted cautiously, as they may primarily reflect numerical dominance rather than epidemiological significance.

Despite these constraints, the data provide original and informative baseline evidence of the local circulation of DENV, *Plasmodium*, and *Leishmania* in Fuente de Oro and Vista Hermosa. These findings highlight the utility of molecular surveillance for detecting pathogen circulation in endemic regions and underscore the need for future studies incorporating species-level identification, tissue-specific analyses, gonotrophic status assessment, and expanded longitudinal sampling to more accurately evaluate vectorial capacity and transmission dynamics. Such efforts will be essential for informing integrated surveillance frameworks and evidence-based vector control strategies in Colombia.

In conclusion, our study underscores the value of entomological surveillance and molecular tools in identifying pathogens circulating locally and driving vector-borne disease (VBD) transmission. Importantly, our results reveal the contribution of species not traditionally considered primary vectors to rural VBD transmission, potentially sustaining ongoing circulation. Integrative approaches that combine mosquito identification with the detection of natural arbovirus and parasite molecular detection may provide a powerful tool for characterizing local VBD transmission dynamics.

## Figures and Tables

**Table 1:** List of primers and Taqman probes used in PCR for the detection of DENV serotypes DENV1–4, CHIKV, ZIKV, OROV, *Plasmodium* y *Leishmania*.

Organism	Primer Probe	Sequence (5′-3′)	Genomic Position	Amplicon Size	Genome Region	Ref.
DENV	DENV-F	TCAATATGCTGAAACGCGHGAGAAACCG	134			[25]
DENV1	DENV1-R	CCCGTAACACTTTGATCGCT	322	208 pb	C-prM	
					
DENV2	DENV2-R	CGCCACAAGGGCCATGAACAGTTT	232	119 pb		
DENV3	DENV3-R	TAACATCATCATGAGACAGAGC	400	288 pb		
DENV4	DENV4-R	TTCTCCCGTTCAGGATGTTC	374	260 pb		
*Plasmodium*	rPLU5	CTTGTTGTTGCCTTAAACTTC	-	1200 pb		[26]
	rPLU6	TTAAAATTGTTGCAGTTAAAACG	-			
*P. falciparum*	rFAL1	TTAAACTGGTTTGGGAAAACCAAATATATT	-	205 pb		
	rFAL2	ACACAATGAACTCAATCATGACTACCCGTC	-			
*P. vivax*	rVIV1	CGCTTCTAGCTTAATCCACATAACTGATAC	-	117 pb		
	rVIV2	ACTTCCAAGCCGAAGCAAAGAAAGTCCTTA	-			
*Leishmania*	18s rDNA F	GTACTGGGGCGTCAGAGGT	-	-	18 s	[27]
	18s rDNA R	TGGGTGTCATCGTTTGCAG	-			
	18s rDNA-P	FAM AATTCTTAGACCGCACCAAG- BHQ1	-			
CHIKV	CHIKV-F	RAAGGAGTGCCGGAARGACAT	1261–1439	178 pb	nsP1	[28]
	CHIKV-R	GACAACCCGGACGACCACAG				
	CHIKV-P	FAM-GARAAGCTYYTGGGGGTCAGAGA-BHQ				
ZIKV	ZIKV-F	AARTACACATACCARAACAAAGTGGT	9271–9373	102 pb	NS5	[29]
	ZIKV-R	TCCRCTCCCYCTYTGGTCTTG				
	ZIKV-P	FAM-CTYAGACCAGCTGAAR-BBQ				
OROV	OROV-F	GACAAGTGCTCAATGCTKGTGT	92–265	173 pb		[30]
	OROV-R	CGTTGTCCGGSACTGGATT				
	OROV-P	TGGTTGACCTTACTTTTRGTGGGGT				

**Table 2:** Frequency of pools collected by genus and by each municipality sampled (Fuente de Oro and Vista Hermosa). Calculated MIR value for each DENV serotype by mosquito genus. Numbers in parentheses refer to percentage per pool.

	*Municipality*				*MIR*			
	Fuente de Oro	Vista Hermosa	Total Pool	Total Specimens	DENV1	DENV2	DENV3	DENV4
*Aedes*	2(4)	48(96)	50	270	62.96	7.41	-	-
*Anopheles*	-	3	3	17	-	-	-	-
*Coquilletidia*	2	-	2	7	-	-	142.86	-
*Culex*	72(87.8)	10(12.2)	82	404	51.98	37.13	12.38	2.48
*Limatus*	-	1	1	8	-	-	-	-
*Lutzomyia*	-	7	7	43	-	-	-	-
*Mansonia*	1	-	1	2	-	-	-	-
*Psorophora*	6(50)	6(50)	12	39	25.64	-	25.64	-

## Data Availability

Data is contained within the article or Supplementary Materials. The original contributions presented in this study are included in the article/Supplementary Materials. Further inquiries can be directed to the corresponding authors.

## Supplementary Materials

The following supporting information can be downloaded at: https://www.mdpi.com/article/10.3390/epidemiologia7030076/s1, Table S1: Collection information and insect pools.
